# A telephone assessment and advice service within an ED physiotherapy clinic: a single-site quality improvement cohort study

**DOI:** 10.1186/s40945-020-00098-4

**Published:** 2021-02-08

**Authors:** Marie Kelly, Anna Higgins, Adrian Murphy, Karen McCreesh

**Affiliations:** 1 Department of Physiotherapy, Mercy University Hospital, Grenville Place, Cork, T12 WE28 Ireland; 2 Emergency Department, Mercy University Hospital, Cork, Ireland; 3 School of Allied Health, Ageing Research Centre, Health Research Institute, University of Limerick, Limerick, Ireland

**Keywords:** Musculoskeletal, Non-attendance, Telephone triage, Timely access, Satisfaction

## Abstract

**Background:**

In response to issues with timely access and high non-attendance rates for Emergency Department (ED) physiotherapy, a telephone assessment and advice service was evaluated as part of a quality improvement project. This telehealth option requires minimal resources, with the added benefit of allowing the healthcare professional streamline care. A primary aim was to investigate whether this service model can reduce wait times and non-attendance rates, compared to usual care. A secondary aim was to evaluate service user acceptability.

**Methods:**

This was a single-site quality improvement cohort study that compares data on wait time to first physiotherapy contact, non-attendance rates and participant satisfaction between patients that opted for a service based on initial telephone assessment and advice, versus routine face-to-face appointments. 116 patients were referred for ED physiotherapy over the 3-month pilot at the ED and out-patient physiotherapy department, XMercy University Hospital, Cork, Ireland. 91 patients (78%) opted for the telephone assessment and advice service, with 40% (*n*=36) contacting the service. 25 patients (22%) opted for the face-to-face service. Data on wait time and non-attendance rates was gathered using the hospital data reporting system. Satisfaction data was collected on discharge using a satisfaction survey adapted from the General Practice Assessment Questionnaire. Independent-samples t-test or Mann Whitney U Test was utilised depending on the distribution of the data. For categorical data, Chi-Square tests were performed. A level of significance of *p* ≤ 0.05 was set for this study.

**Results:**

Those that contacted the telephone assessment and advice service had a significantly reduced wait time (median 6 days; 3–8 days) compared to those that opted for usual care (median 35 days; 19–39 days) (*p* ≤ 0.05). There was no significant between-group differences for non-attendance rates or satisfaction.

**Conclusion:**

A telephone assessment and advice service may be useful in minimising delays for advice for those referred to ED Physiotherapy for musculoskeleltal problems. This telehealth option appears to be broadly acceptable and since it can be introduced rapidly, it may be helpful in triaging referrals and minimising face-to-face consultations, in line with COVID-19 recommendations. However, a large scale randomised controlled trial is warranted to confirm these findings.

**Supplementary Information:**

The online version contains supplementary material available at 10.1186/s40945-020-00098-4.

## Introduction

Musculoskeletal conditions make the most significant contribution to the global burden of disability, with more than 20% of the world’s population living with a painful musculoskeletal disorder [1]. Given our aging population [2], the burden of these conditions is expected to increase, placing further demands on limited healthcare resources. Emergency departments (EDs) are one of the main providers of treatment for musculoskeletal conditions, particularly non-traumatic neck and back pain, with early access to physiotherapy strongly advocated for within the Irish Health Service Executive National Emergency Medicine Programme [3]. Physiotherapy intervention within a five-day period following injury significantly reduces work absenteeism [4], and consequently, has economic benefits, with musculoskeletal conditions ranked as the second largest cause of days lost from work [5]. However, timely access to physiotherapy is often an issue, with waiting lists for treatment of several months in some regions [6] which is likely to result in adverse effects on health outcomes and increased healthcare utilisation for patients with musculoskeletal conditions [7]. Furthermore, delayed access to physiotherapy can lead to increased non-attendance rates, with many not attending appointments when they are finally offered one [8]. This, together with the fact that those that gain minimal or no benefit from physiotherapy might have benefited more if they have been reviewed more quickly [9, 10] clearly illustrate that a significant amount of physiotherapy services are utilised ineffectively and inefficiently [10].

Telehealth, a subcategory of eHealth [11], is becoming increasing popular in an attempt to meet these challenges. Clinicians are utilizing innovative methods of delivering care, including telephone consulting, with physiotherapy-led telephone assessment and advice services established across many regions such as the UK [12] and Australia [13]. Typically, within a telephone advice and assessment service, service users are invited to telephone a senior physiotherapist for initial assessment and advice, which is followed up with posted relevant self-management resources and exercise leaflets. Alternatively, face-to-face consultations are arranged if deemed necessary following the initial telephone assessment or if the patient’s symptoms are not resolving after the initial advice [8]. In line with best practice, the focus of all consultations encompasses the exclusion of potentially serious pathology and as indicated appropriate onward referral [14] This service model is in keeping with recommendations from physiotherapy associations worldwide due to the COVID-19 pandemic, that the majority of appointments are conducted remotely, minimising face-to-face sessions where possible [14].

Although robust research is lacking on the role of telephone assessment within the field of physiotherapy, evidence exists on the safety, clinical- and cost-effectiveness, along with patient acceptability within other clinical settings such as nurse telephone consultation for routine asthma review and in out of hours primary care [15–18]. The only high quality randomized controlled trial within physiotherapy to evaluate a telephone triage service (‘PhysioDirect’) was conducted within a primary care setting, reporting that the service was as clinically effective as usual face-to-face care, with regards to participants’ physical functioning [12]. Shorter waiting times and reductions in non-attendance rates were also illustrated. Furthermore, a nested qualitative study [19] concluded that a telephone assessment and advice service was broadly acceptable to participants, due to more timely access to advice.

This patient care pathway reflects the evidence about the effectiveness of different modalities within physiotherapy for various conditions. For example, trials have found that a single session of advice from a physiotherapist is as effective as a course of physiotherapy for patients with back pain [20, 21], with research also advocating a single physiotherapy advice session for those with persistent acute whiplash symptoms [22]. Furthermore, physiotherapy-led advice and exercise are effective in knee pain [23–25]. Alternatively, for other presentations such as shoulder and neck pain, evidence exists suggesting that manual therapy as an adjunct to advice and exercise is more effective than exercise and advice alone [26–28]. Therefore, a care pathway, which provides assessment, advice and triage initially, while reserving more intensive (and expensive) treatments for those who do not improve, may be the most cost-effective strategy. This care pathway would also limit face-to-face consultations, in line with COVID-19 related recommendations [14]. However, to date this model of service delivery has yet to be evaluated within either the Irish healthcare system or physiotherapy ED setting. Therefore, the main objective of this study was to evaluate whether a telephone assessment and advice service can reduce the wait time and non-attendance rate for physiotherapy compared to the usual care pathway. A secondary aim was to evaluate whether a telephone assessment and advice service is acceptable and satisfactory to service users.

## Methods

### Study design

This study was a single-site cohort study with two parallel groups with recruitment between May and August 2018. Data collection was complete in May 2019. The comparison was between patients that opted for a service based on initial telephone assessment and advice, versus routine face-to-face appointments. This study design was utilised due to a consistently high non-attendance rate (approx. Average 30%) and some qualitative research nested within the ‘PhysioDirect’ study [19] indicating that telephone assessment and advice services are best placed alongside face-to-face services rather than as a replacement. This study was approved by the Clinical Research Ethics Committee of the X, Ireland and carried out in the ED and outpatient physiotherapy department at X Hospital, X, Ireland. All participants provided signed informed consent to participate in this study, which was carried out in accordance with the Declaration of Helsinki. The STROBE standardised reporting guidelines were followed in the reporting of this research [29] (Additional File 1).

### Participants

All adults (aged ≥ 18 years of age) were invited to participate in this study, if following their attendance at the X Hospital ED, physiotherapy was deemed appropriate by a member of the ED team (Consultant, Non-Consultant Hospital Doctor or Advanced Nurse Practitioner). Inclusion criteria were deliberately broad to maximize generalizability. Participants were excluded if they were unable to communicate in English via telephone or were referred with non-musculoskeletal problems.

### Procedures

All eligible participants were provided with a participant information leaflet in ED and the two treatment pathways were discussed by a member of the ED team, with the patient choosing based on their preference. The first treatment option was the physiotherapy telephone assessment and advice service, while the alternative was the usual care pathway i.e. appointment made for a face-to-face consultation. Patients that opted for the telephone assessment and advice service had their verbal consent noted during the first telephone consultation with another copy of the participant information leaflet, questionnaires, consent form and prepaid return envelope sent out in the post on discharge. Those who did not respond to the first mail out were sent a second mail-out approx. Two weeks later. Those that opted for a face-to-face consultation provided written informed consent during the first consultation if they wished to participate.

#### The telephone assessment and advice service

Patients were invited to telephone a senior physiotherapist at specific times for initial assessment and advice. Generally, at the end of the consultation, the senior physiotherapist posted a relevant advice leaflet about exercises and self-management to the patient and invited them to phone back in approx 2–4 weeks to report progress if appropriate. At that point, they were given further advice or booked for a face-to-face appointment if necessary. If the initial call indicated more urgent face-to-face care was required, this was booked at the outset.

#### Usual care pathway

Usual care generally involved an initial face-to-face physiotherapy assessment and then a series of follow-up treatment appointments over several weeks or months, according to therapist’s discretion.

#### Data collection

To characterize the study population, demographic information such as employment status, location of symptoms, age, gender etc. was recorded on a data collection form.

#### Outcome measures

The primary outcome measures were wait time to first physiotherapy contact, non-attendance rates and participant satisfaction. Wait time to first physiotherapy contact and non-attendance rates (defined as ratio of number of missed appointments to total number of scheduled appointments) was gathered using the hospital data reporting system, Implement Single Patient Administration System (iPIMS). Satisfaction data was collected using a satisfaction survey adapted from the General Practice Assessment Questionnaire, which has been utilised previously [12], with internal reliability confirmed using rotated factor analysis. Overall satisfaction with the service was based on one question. All questions use six point Likert scales. To characterize clinical outcome on the last physiotherapy appointment, both groups were asked one question either face-to-face or via telephone, about overall improvement in the main problem for which the patient was referred to physiotherapy (global improvement score – a seven point scale from “very much better” to “very much worse”). This was chosen as no disease specific measure would be appropriate for this study, given the varied range of musculoskeletal conditions referred to physiotherapy via ED.

#### Statistical analysis

All data analysis was undertaken using the Statistical Package for the Social Sciences Version (SPSS) 23.0 [30]. A level of significance of *p* ≤ 0.05 was set for this study. Normality of the continuous variables was tested with the Shapiro-Wilk test and appropriate descriptive statistics were calculated. Where the normality assumption was violated, equivalent non-parametric tests were used. The Mann Whitney U Test was utilized to evaluate between-group differences in wait time and number of physiotherapy consultations given the non-normal distribution. Median and inter- quartile values (Q1 – Q3) are presented as these values are better represented by the median rather than the mean, with the median less sensitive to outliers [31]. Chi-Square tests were performed to evaluate between group differences for the categorical data (non-attendance rate, satisfaction and global improvement scores). Within this quality improvement project, an integral feature is that patient preference dictates the chosen care pathway. This, together with some literature indicating that uncertainty exists in power calculations for time series analysis [32, 33], meant that a priori power calculation was not performed.

## Results

### Participant flow and recruitment

**Fig. 1:**
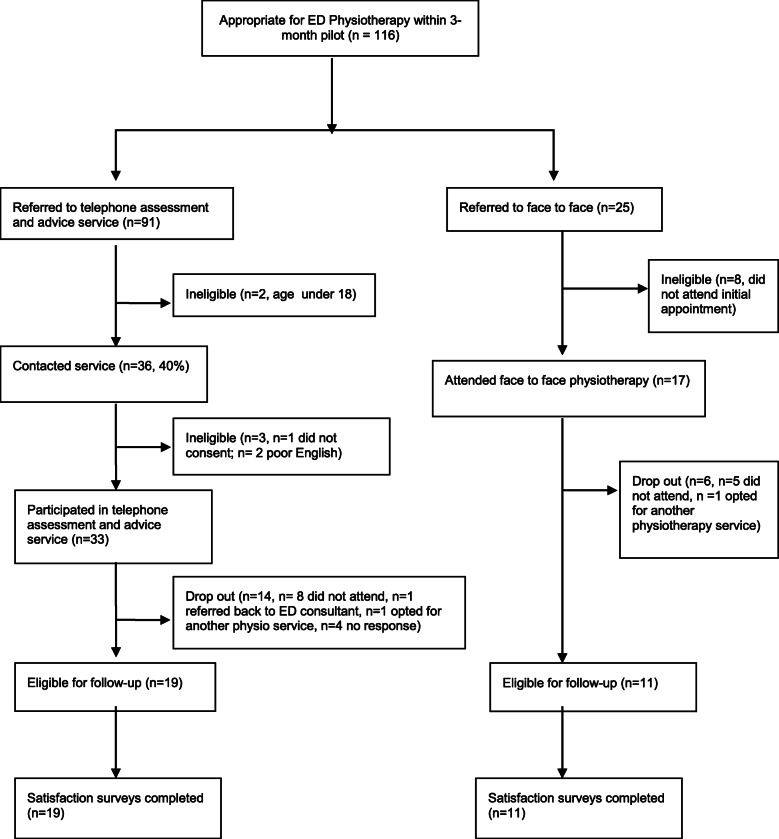
Flow of Participants through the study

Figure 1 illustrates the flow of participants during the study. Of 116 patients deemed suitable for ED Physiotherapy, 78% (*n*=91) opted for the telephone assessment and advice service. Of those deemed eligible at that stage, 40% (*n*=36) contacted the service; however three participants were excluded (*n*=1 did not consent; *n*=2 poor English). Table 1 illustrates baseline demographic and clinical characteristics for each group.

**Table 1:** Participant demographics

	No (%) of patients	
	Usual care (*n*=17, 17%)	Telephone service (*n*=86, 83%)
Female sex	6 (35%)	37 (41%)
Age (years) ^a^	41 (36–66)	43 (31–56)
Employed^b^	9 (53%)	18 (21%)
Site of musculoskeletal problem^b^		
Cervical	3 (18%)	5 (6%)
Thoracic	0	1 (1%)
Lumbar	1 (6%)	19 (22%)
Upper Limb	7 (41%)	15 (17%)
Lower Limb	6 (35%)	42 (49%)
Multiple	0	4 (5%)
Duration of symptoms (weeks)^ab^	6 (5–8)	4 (2–16)
New presentation^b^	14 (82%)	20 (61%)
Recurrent presentation^b^	3 (18%)	13 (39%)

^a^Median (interquartile range)

^b^Excludes those that did not opt in/attend initial appointment

### Primary and secondary outcomes

Those that contacted the telephone assessment and advice service had a significantly reduced wait time for consultation (median 6 days; 3–8 days) compared to those that opted for the face to face care pathway (median 35 days; 19–39 days) (*p* ≤ 0.05).

For the telephone advice and assessment group, there was 99 appointments in total, with 10 ‘did-not-attends’ at subsequent face to face appointments, resulting in a 10% non-attendance rate. For the usual care group, there was 68 appointments in total, with 15 ‘did-not-attends’, resulting in a 22% non-attendance rate. This difference was non-significant between both groups (*Χ*^2^(2) = 4.41, *p* > 0.05).

**Fig. 2:**
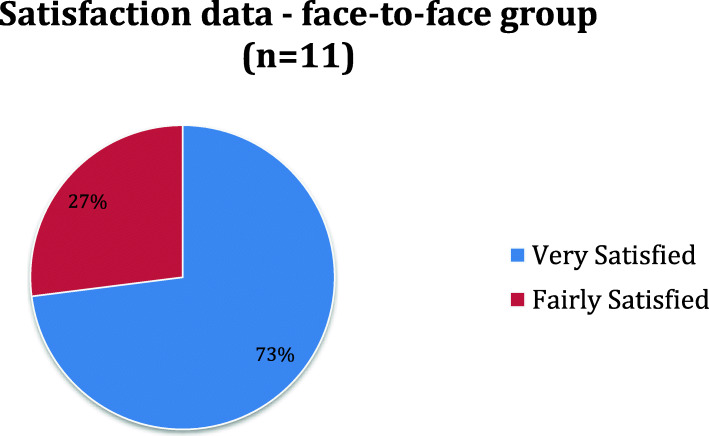
Satisfaction data results for face-to-face group (*n*=11)

**Fig. 3:**
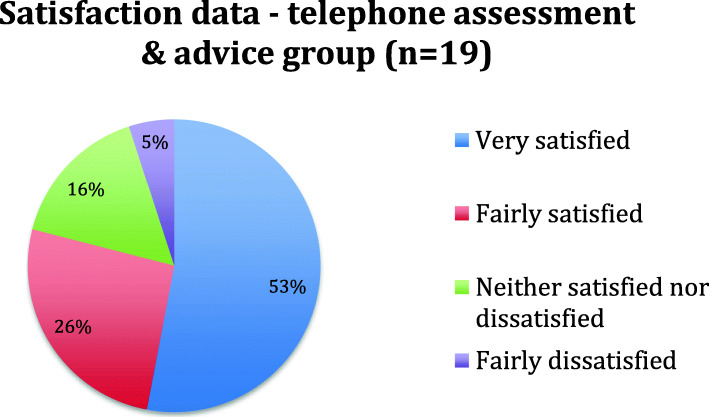
Satisfaction data results for telephone assessment & advice service (*n*=19)

There was no statistically significant difference between groups with regards to overall satisfaction (*Χ*^2^(3) = 3.44, *p* > 0.05) (Figs. 2 and 3).

**Fig. 4:**
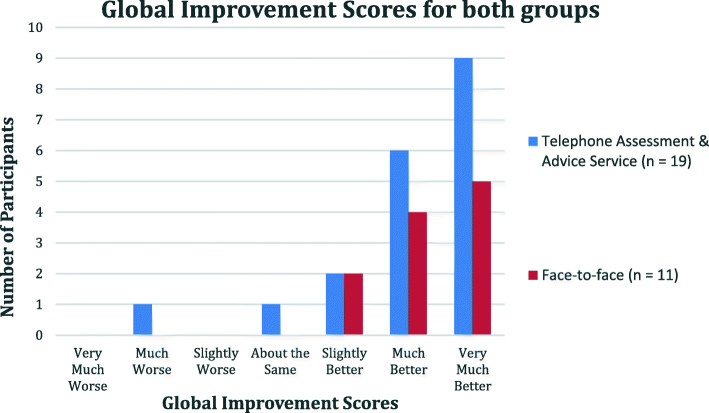
Global improvement scores for both groups

There was no statistically significant difference between groups with regards to global improvement scores (*Χ*^2^(4) = 3.00, *p* > 0.05) (Fig. 4).

### Process of care

Of the 33 eligible participants that contacted the telephone assessment and advice service, 14 (43%) were managed entirely by telephone consultation. Patients in the telephone assessment and advice service had a similar number of physiotherapy contacts overall (via telephone and face to face) (median 2; 1–4) compared to the usual care group (median 2; 1–5) (p > 0.05). No adverse events were reported in either the telephone assessment and advice service or face-to-face group.

## Discussion

The purpose of this quality improvement cohort study was to evaluate whether a telephone assessment and advice service could reduce non-attendance rates and improve wait times within an ED Physiotherapy clinic. While text message [34] and telephone reminders [35, 36] have proven effective at reducing non-attendance rates elsewhere, these resources are not currently available within this clinic. A telephone advice and assessment service requires minimal resources, with the added benefit of allowing the healthcare professional streamline care. Given the importance of access to musculoskeletal physiotherapy within the ED setting and the impact of high non-attendance rates on patients and healthcare resources, it is imperative that strategies that may improve waiting times and non-attendance rates are evaluated.

The main finding from this cohort study is that, compared with usual face-to-face care, the telephone assessment and advice service care provided faster access to ED physiotherapy without compromising on service user satisfaction. While no studies to date have assessed the impact of telehealth on wait times within an ED Physiotherapy clinic, these findings are consistent with research conducted within other physiotherapy settings [12, 37], along with other healthcare settings and patient populations [38–41]. No significant reduction in the non-attendance rate was observed for this telehealth care pathway, although it is worth noting that the 10% (10/99) non-attendance rate observed in the telephone assessment and advice group compares well with regards to national [42] and international figures [43, 44]. This non-significant finding is in sharp contrast to other literature [12, 39, 41, 45–47] and may be partly explained by the methodological study design and imbalance between study groups resulting in a high risk of confounding bias. Predictors of non-attendance include gender (male), younger age, unmarried status, low educational level and receipt of long-term welfare payments [48]. While data was collected on a number of these variables, the sample size, particularly in the face-to-face group is insufficient to control for these confounding factors using statistical methods. This quality improvement project was designed in a patient-centred manner with patients making an informed decision with regards to their care pathway preference and while this resulted in an imbalance between study groups, this approach was integral to the project [49]. Furthermore, the design of this quality improvement project was informed by qualitative research findings indicating that a telephone assessment and advice service may be most suitable as one method of accessing physiotherapy services, rather than as a replacement of face-to-face care pathways [19].

This telephone assessment and advice service operated via a ‘one-way’ system in general, where the senior physiotherapist waited for patients to call at specified times on a Monday, Wednesday and Friday morning. These times were chosen based on previous service provision and to minimise unnecessary delays to service access as much possible. Nevertheless, the median wait time was 6 days, in line with other literature evaluating a musculoskeletal physiotherapy telephone service, operating via a ‘one-way’ system [12, 50]. The non-attendance rate of 22% (15/68) in the face-to-face group is not surprising since longer waiting times are associated with higher non-attendance rates [51]. The median wait time of 35 days for those within the usual care group is in line with other hospital outpatient physiotherapy waiting time figures in Canada [52, 53] and the UK [54].

Findings from this study suggest that more than a third of those suitable for an ED Physiotherapy service can be managed via telephone consultation alone. This figure is somewhat lower than figures reported in other studies (47–50%) [12, 15] although it is worth noting that these studies were conducted within a primary care setting and this may partially explain this discrepancy. In some instances within an ED physiotherapy setting, face-to-face consultations may be indicated to comprehensively screen for potentially serious pathology with a patients’ clincial presentation not always falling into a clear diagnositc category [14]. Furthermore, while a high level of agreement between telehealth (specifically videoconferencing) and face-to-face assessments has been demonstrated [55], this study involved participants with chronic musculoskeletal conditions. Hence, this finding may not be generalisable to a telephone ED Physiotherapy service which commonly encounters acute and sub-acute musculoskeletal presentations. Limited response to the telephone-delivered intervention or patient preferences are other reasons why a face-to-face consultation was indicated.

It is worth noting that 78% (91/116) opted for the telephone assessment and advice service suggesting this eHealth solution is broadly acceptable to patients. Furthermore, there was no significant difference between groups with respect to the satisfaction survey results, with both groups demonstrating a similar response rate. This is consistent with qualitative findings from the PhysioDirect trial [19] along with systematic review evidence from general practices [56] but in contrast to findings from the ‘PhysioDirect’ quantitative evaluation [12]. One explanation for this may be methodological design, with our telephone assessment and advice service operating alongside a usual face-to-face care pathway, with patients choosing based on their preference.

This study utilised a global improvement score to crudely characterise clinical effectiveness, with no significant differences observed between groups. While this measure has good psychometric properties across a broad range of musculoskeletal conditions [57], it is insufficient to comprehensively evaluate this multidimensional concept. Another possible explanation may be that for a number of conditions such as osteoarthritis and back pain, evidence-based guidelines [58, 59] recommends advice about maintaining physical activity levels, structured exercise therapy and self-management interventions rather than manual therapy. Recent evidence suggests these interventions can be effectively delivered remotely [13, 60, 61] and hence, perhaps these patients have little more to gain from an episode of face-to-face care.

A number of limitations are acknowledged, with the high risk of confounding bias foremost. Secondly, a large proportion (60% or 55/91) that opted for the telephone assessment and advice service did not contact the service. While the ‘did not contact’ figure is similar to figures reported elsewhere [12], we did not capture reasons for non-contact or non-attendance for those that did initially engage with either pathway. The sample size was also small with an imbalance between groups, and this in conjunction with the observational nature of the study highlights the need for future research.

## Conclusion

In conclusion, this study indicates that a telephone assessment and advice service may be a useful strategy of reducing delays for advice for musculoskeletal problems for patients referred by a member of an ED team for ED Physiotherapy. Furthermore, this eHealth option appeared to be broadly acceptable to patients with musculoskeletal disorders. However, further research involving a larger sample size and utilising a randomised controlled trial design is warranted to validate these findings.

## Supplementary Information

**Additional file 1.**

## Data Availability

The dataset of the current study is available from the corresponding author.

## Abbreviations

ED: Emergency Department
iPIMS: Implement Single Patient Administration System
SPSS: Statistical Package for the Social Sciences Version
